# The Pathology and Treatment of Cholera

**Published:** 1866-05

**Authors:** Henry M. Lyman

**Affiliations:** Pathologist to the Cook County Hospital


*The Pathology and Treatment of Cholera.* By HENRY M. LYMAN, M. D., Pathologist to the Cook County Hospital.

(Read before the Chicago Medical Society, March 30, 1866.)

In this essay I can claim nothing that is new. Resulting from an independent effort to examine the phenomena of cholera in the light of physiology and pathology, I am not surprised, but am pleased to find that my conclusions have been anticipated by others, to whose writings my attention has been directed since the completion of this study. "There is nothing new that is good; nothing good that is new."

For the sake of clearness in argument, I shall assume that the symptoms of cholera are produced by the action of a positive agent which operates in the blood, vitiating its fluid portion and exciting the system through the fibres of the ganglionic nerves, to an effort for the elimination of the intrusive poison.

That cholera is essentially a disease which consists in contamination of the blood, seems to be indicated by all its symptoms. Vomiting, purging, sweating, cramps, collapse and reaction are phenomena characteristic of elimination, whether they be caused by an over-dose of podophyllin, or by a draught of cholera poison. These are not vague and purposeless efforts, but are possessed of a significance that is most instructive.*

* The analogy which exists between the phenomena of all diseases should also be kept in mind. In pneumonia the exudation which is poured into the air-cells affords vital relief by emptying the distended capillaries. The mechanical difficulties which grow out of this exudation are probably less prejudicial than the consequences which would be manifested, were exudation from the vessels during inflammation rendered impossible by any cause.

I think we need not hesitate to affirm the proposition that the symptoms of cholera are caused by the violent efforts of the system to rid itself as speedily and as effectually as possible of the fluid portion of the blood.* In addition to the phenomena occasioned by this eliminative effort, must be reckoned the secondary and consecutive phenomena arising from an arrest of the functions of nutrition, secretion and excretion, which is unavoidable under the conditions most favorable to such an extraordinary method of elimination.

Prof. N. S. Davis has fully shown how caloric acts as a predisposing cause of watery effusions from the blood, by depressing the integral vitality of the tissues, and by producing a general laxity highly favorable to perspiration and to intestinal flux. The action of caloric alone, however, will not suffice to produce the disease in question. In cholera morbus, bilious diarrhœa and Asiatic cholera, the symptoms of the disease do not manifest themselves until the blood has become vitiated by the introduction of some foreign agent, and by the retention of excrementitious substances. To eliminate this *materies morbi* is, then, the effort of nature; and the manner in which the process is effected, is an evidence of the wonderful skill with which the animal organization is contrived. A temporary derangement of the circulation is made the means of purifying the blood. In a state of health the tonicity of the blood-vessels is maintained, and their calibre is regulated by the action of the branches of the ganglionic nerves which are distributed to their walls. Under the influence of the depressing causes which, during the prevalence of certain atmospheric conditions, tend to diminish the integral vitality of all parts of the system, these ganglionic nerves undoubtedly suffer with the rest of the tissues. Now it is well known that debility is the parent of morbid susceptibility and morbid irritability. During health the passage of healthy blood excites these nerves to a degree that influences the walls of the blood-vessels sufficiently to maintain that degree of tonicity which

* That the poison resides in the serum, is rendered probable by the fact that it is the watery portion of the blood rather than its solid constituents, which is ejected from the system. In yellow fever the red globules are affected, as appears from the amount of hematoidine in the evacuations.

is most subservient to healthy circulation. But when, as a consequence of the impaired nutrition, secretion and excretion which result from a diminution of integral vitality, or when, as a consequence of the intrusion of a poison from without, the blood becomes vitiated, it fails to communicate to the ganglionic nerves that healthy stimulus which is essential to the proper calibre of the blood-vessels. Subsequent phenomena indicate that one result of this morbid irritation is a spasmodic action of the muscular coat of the smaller ramifications of the blood-vessels,—a condition of which the effect is naturally more conspicuous in the arterioles than in the veinules, because of the superior muscular organization of the arteries. That this spasmodic muscular action is perfectly compatible with debility, is proved by the powerful muscular contractions which occur in cholera and in tetanus, in delirium tremens, and in many other diseases where vitality is at the lowest ebb. In addition to this, in cholera the vigor of the heart is diminished by the failure of the innervation derived from the pneumogastric nerve, a circumstance which is proved by the loss of the voice, and the acceleration of the movements of the heart which occur during the disease;—precisely the effects produced by section of the pneumogastric nerve at its origin.

Having then a condition characterized by arterial debility with consequent irritability and diminished cardiac vigor, the blood is forced with less power from the heart, so that it moves in smaller quantity from the right to the left side of the heart, and the venous system becomes unusually gorged with blood. When this derangement is not excessive, the over-loaded veins of the bowels obtain relief by moderate exudation into the alimentary canal. This is the stage of diarrhœa, known, in the absence of specific causes, as bilious diarrhœa. When the system is laboring under the influence of cholera poison, this stage of the disease is known as *cholerine*. It is essentially a recuperative effort, and tends to promote the relief of the patient. But when the amount or the intensity of the poison is too great for elimination by this gentle process, more violent measures become necessary. Responding to the increased irritation produced by the circulation of vitiated blood, the arterioles urge on the blood more effectually, while the heart, more completely deprived of the influence of the pneumogastric nerves, and depressed by over-distention, beats more frequently and with less vigor. The veins are not emptied as they should be in health, partly because of the diminished strength of the heart, and partly because the current of blood is materially obstructed in its passage to the lungs. That this is not a mere matter of theory, is shown by examination of the bodies of those who die of cholera before the stage of reaction occurs.* The system seems intolerant of the blood, and refusing to receive it, attempts to confine the fluid within the venous cavities, where, physically speaking, it is the farthest removed from the tissues of the body. At the same time so greatly is vitality depressed, that the walls of the venous capillaries and venules can no longer resist the pressure of their contents, and the watery portion of the blood is poured forth upon the surfaces within and without the body. Extending its influence to the spinal nerves, the morbific agent excites muscular contractions which serve to force towards the central cavities, the blood from the deeper and more distant portions, where it would otherwise linger in the capillaries and more certainly poison the system.† Contraction of a kindred nature occasions the expulsion of the fluids that are poured into the alimentary canal. Hence, the vomiting, purging, sweating and cramping which characterize this stage of the disease. At the same time the functions of the liver and kidneys are impeded; not because their continuance would be prejudicial, but because, having been designed for the performance of certain functions under the ordinary conditions of health, it is mechanically impossible for these organs to continue their operations under those conditions

*The lungs are so bloodless that Dr. Metcalfe, of New York, regards this as the cause of the coldness of the breath of a patient in collapse. This is partly true, but the principal cause of the diminution of temperature is to be found in the suspension of cell-activity throughout the system, which occurs in the presence of the poison which causes the disease. As soon as this poison has been sufficiently eliminated, the cells resume their activity, and the temperature of the body rises, even for a considerable time after the death of the patient.

†This statement accords with the current opinion relative to the production of muscular motion. The more rational views of the causation of muscular action, advanced by C. B. Radcliffe in his lectures on pain, paralysis and epilepsy, serve to explain the phenomena of cramp in cholera in a manner much more consonant with the ideas which have been proposed above. He has shown that convulsions, cramps, and muscular motion in general, can be produced by impeding the supply of arterial blood, by the circulation of vitiated blo d, (which is equivalent to the arrest of arterial supply,) and by a diminution of nervous influence;—three conditions which are present in cholera.

of intense venous congestion which are most favorable to the speedy elimination of the cholera poison. As the effect of this agent becomes more fully developed, the exclusion of the blood from the tissues is made more perfect by the more violent operation of the causes which have been described. The patient becomes livid, pulseless, shrivelled, collapsed. The blood can with difficulty reach the lungs, and the small quantity which finds its way to the left side of the heart is hurried back into the venous cavities, where it is least innocuous to the system. Thus the venous pressure is maintained until a sufficient portion of the vitiated fluid has been ejected from the body. If the vital powers of the system have been too far overcome in this process, death may occur at this stage; but if the vitality of the tissues still remains, reaction now begins, and its progress and completeness depend, not only upon the degree of unimpaired energy, but upon the extent of the injury which may have resulted from the accumulation of excrementitious matters during the suspension of the hepatic and renal functions. Relieved in great measure of the irritating fluids which have excited arterial spasm, the heart receives new innervation from the pneumogastric nerves, and the walls of the arterioles become relaxed even beyond their natural calibre. Circulation is restored; the veins are emptied sufficiently to arrest the morbid transudation of their contents; secretion is renewed, and recovery commences. The subsequent fevers, congestions and inflammations which so often delay or prevent recovery after the establishment of reaction, are only the natural consequences of the mechanical violence to which the system has been subjected by the choleraic paroxysm, associated with imperfect or excessive reaction, and the impaired nutrition resulting from the vitiated condition of the blood. These sequelæ of the disease need not be considered any farther in this connection.

Such then, is the theory of cholera. In opposition to this theory are urged two objections, which at first might seem to carry some weight, but which are easily set aside by a reference to the teachings of physiology.

Against the theory of a poison acting through the watery portion of the blood it is objected that, if the vitiation of this fluid is the cause of the disease, and if the poison is really eliminated by the evacuations which occur, the severity of the symptoms of the disease should steadily decline from the commencement of the evacuations. Now we have through the whole argument assumed that the manifestations of the disease are produced through the immediate effect of the poison upon the ganglionic nerves; and as it is an established fact that "the effects of stimulation or irritation of this system are not instantaneously manifested, as is the case in the cerebro-spinal system, but are developed slowly and gradually," a little reflection will obviate the difficulties which at first sight are presented by the spectacle of a proximate cause acting with increasing energy, while the remote cause is becoming continually more inert.

The second objection is still more weighty, though far from conclusive. It is urged that the phenomena of collapse are due to an arrest of the circulation in consequence of the viscidity of the blood, which has been drained of its watery portion, and that these phenomena cannot be attributed to spasm of the pulmonary arterioles, because there is not a degree of dyspnœa sufficient to warrant a belief in the existence of any pulmonary obstruction. Now collapse is not simply the result of impeded circulation, else it would not differ from syncope. It results from a depression of the vitality of the system, combined with an arrest of the circulation. The specific consequences of such an arrest would be the same, whatever its cause; and were it not for its relation to the plan of treatment, the exact nature of the impediment which undoubtedly exists would be a matter of no importance. That simple viscidity is not the cause of the impeded circulation, is rendered clear by the fact that after reaction has taken place, the blood moves without difficulty, even before its volume has been restored; nor can the sudden occurrence of the collapse which occasionally supervenes before the manifestation of other grave symptoms be explained by the supposition of a morbid viscidity of the blood. In cases of this class there is little evacuation; collapse is suddenly declared; the patient experiences a want of air, and suffers indescribable agony; death speedily closes the scene, while the blood is still fluid, before the strength has been exhausted by suffering and evacuation. These phenomena bear a striking resemblance to the phenomena which follow the admisssion of air into the right side of the heart,—an accident which produces distress and speedy death by a mechanical impediment to the passage of blood from the right ventricle to the pulmonary capillaries.

As for the dyspnœa which some think should result from such an obstruction to the current of the blood, it is sufficient to remember that the phenomena of asphyxia are due to a cessation of the supply of oxygen to organized tissues in which the processes of nutrition are still active. Whenever the processes of nutrition are partially suspended, the supply of oxygen may be almost entirely cut off, without exciting that distress which would be felt as a consequence of such an arrest during a state of health. The following experiment performed by Bernard, illustrates this proposition: "A sparrow was confined under a bell-glass for one hour and a half, at the end of which time another was introduced; the first being still comparatively vigorous. The second became instantly much distressed, and died in five minutes; but ten minutes after, the sparrow which had been confined for more than an hour and a half, was released, and flew away." The bird which was first imprisoned, gradually poisoned the air of the receiver, by absorbing oxygen and exhaling carbonic acid gas. By this process the oxygenation of the blood was progressively hindered, yet no symptoms of distress were manifested, because the processes of nutrition were by inhalation of a poisonous atmosphere, correspondingly depressed to such a degree, that the demand for oxygen in the system was insufficient to excite that *besoin de respirer* which was immediately manifested by the second bird, in whose tissues, integral vitality being unimpaired, the processes of nutrition were so slightly depresssed, that the demand for oxygen was greater than could be supplied from the vitiated atmosphere of the receiver. Hence, the symptoms of distress and the speedy dissolution of the stronger and healthier bird—actually destroyed by its own superior vitality.* Now the condition of a cholera patient is very similar to the condition of the bird under a receiver. Gradually deprived of blood through the diminution of the arterial current, and depressed by the overwhelming influence of the cause of the disease, the processes of nutrition are impeded, and the demand for oxygen in the tissues is so slight, that when collapse finally occurs, the only symptoms of asphyxia are sighing, yawning, or a trifling degree of dyspnœa, instead of those symptoms of acute distress, faintness and want of air which attend collapse when it ushers in the disease. The same facts were presented in the case of a patient who recently died of pyæmia, at the County Hospital. In that case the liver exhibited a marked degree of superficial paleness, but, on section, black blood poured from its cavities as freely as if the incision had been carried through living flesh. On opening the thorax, the anæmic condition of the lungs was so remarkable that I expected to find some obstruction to the passage of blood between the liver and the lungs. Section of the heart revealed a sufficient obstruction in the shape of a firm straw-colored clot which filled the right side of the organ, and extended to the ultimate ramifications of the pulmonary artery.† Here was a condition producing appearances analogous to those which characterize the morbid anatomy of cholera during the stages previous to reaction: there was impeded circulation of the vitiated blood, leading to its confinement in the venous cavities, and producing the diarrhœa and sweating which serve to eliminate the *materies morbi*, yet none of the acute distress which would have been inevitable had the processes of nutrition been creating in the tissues a constant demand for oxygenated blood.

It has then been shown that the symptoms of cholera indicate a condition in which the system labors under the effects of a poison which is contained in the watery portion of the blood; that the efforts of nature are directed to the exclusion of this

* Does not this explanation hint at one of the causes of the greater mortality which attends the outbreak of all epidemics of dangerous disease?

† In this case the bloodless appearance of the lungs as they were exposed *in situ* was very remarkable, yet the obstruction was not sufficient to prevent the occurrence of considerable hypostatic congestion. May we not in this way reconcile some of the conflicting statements respecting the condition of the lungs after death in collapse?

vitiated fluid from the tissues, and to the ejection of its poisonous constituents from the body; that the arrest of secretion and the retention of excrementitious matters, are the inevitable result of that derangement of the circulation which is most favorable to the forcible and speedy purification of the blood; and also, that the absence of certain phenomena does not contradict this view, but is really owing to that serious depression of the integral vitality of the tissues which is the true source of danger in the disease. In accordance with this theory, the indications for treatment are: 1st, to administer an antidote to the poison that causes the disease; 2d, to sustain the integral vitality of the system; 3rd, to favor the evacuation of the poisonous constituents of the blood; and 4th, to supply the means for a restoration of a healthy circulation. The antidote to the poison of cholera remains for future discovery, so we must reluctantly forego this branch of the treatment. The attempt to sustain the vital energies of the patient next engages our attention. You are all sufficiently familiar with the methods which are commonly employed for this purpose. I shall in this connection only advert to the use of stimulants, which usually constitutes the chief reliance in efforts to sustain vitality. Since the prostration of cholera consists in the depression of vitality which results from the circulation of poisoned blood, and since the collapse of the disease consists in this depression, with the added effects of an impeded circulation, the condition of the patient is widely removed from that state of syncope which is the result of a temporary debility of the heart. In syncope the blood and the tissues are healthy,—all that is needed is the power of propulsion from the heart. This is furnished by stimulants, and may be sustained by them until the full measure of cardiac vigor is restored. But when the vitality of the tissues is overwhelmed by contact with poisonous blood, an agent which serves not to purify that blood, but only to increase the freedom of its supply by spurring the circulation, cannot fail to be injurious. Alcoholic stimulants, moreover, have the fatal property of diminishing the activity of the processes of disintegration and nutrition, processes which in cholera are already at their lowest ebb. Hence, we should anticipate that the use of diffusable stimulants must be injurious in this disease, and experience fully justifies the anticipation. But, if we possess any agents which can support the vitality of the tissues without too much acceleration of the circulation, their use will be found beneficial.*

The next indication is to favor the evacuation of the poisonous constituents of the blood. This does not at all imply the acceptance of the doctrine, that like cures like, requiring the use of purgatives and emetics. The duty of the physician consists in rightly interpreting the efforts of nature, and in placing the patient under those conditions most favorable to the unimpeded activity of his own natural powers. Hence, it is as important to know what to avoid as to know what to do. It is in the preliminary stages of the disease as important to keep in view the elimination of the poison, as it ever can be in the later stages. While restraining the diarrhœa, which is only dangerous because of its tendencies, we shall not contribute to the safety of the patient if we neglect to promote the activity of the skin, the kidneys and the liver. Hence, the advantage gained by the use of an intestinal astringent like sulphuric acid, which increases the activity of the kidneys, especially when combined with diuretics and with diaphoretics. In the successive stages of the disease this consideration should deter us from the use of those astringents which simply prevent the escape of the fluids of the blood. The worst cases are by no means those which are characterized by the most copious evacuations. It will not often be necessary to use evacuant remedies, but when collapse occurs suddenly without any antecedent discharge, diaphoretics, emetics and even purgatives are required. So long as we have no antidote to the poison, nothing can be more calculated to endanger the safety of the patient than an attempt to oppose its elimination from the system; consequently, when the processes by which the blood is ordinarily purified are necessarily suspended, the attempt to arrest purging by the use of opium and astringent remedies can, if

* The good effects of *small* doses of laudanum in collapse are well known, and explain the success which has attended the use of the celebrated cholera mixture employed in Constantinople.

successful, only add to the dangers which surround the sufferer.* The use of calomel is defended on the ground that it is the most powerful agent which can be employed to increase the discharge of bile. True enough, and its use is indicated in the earlier stages of the disease before the appearance of alarming symptoms, while there is only inactivity of the hepatic function. But when the liver has become so deeply engorged with blood that secretion is difficult if not impossible, what benefit can accrue from the use of such an agent? Depletion is needed to restore secretion in this stage. Good results have without doubt sometimes followed the administration of calomel during the stage of prostration, but they seem to have been confined to that diminution of gastric irritability which results from the local effects of the drug upon the mucous surface of the stomach. Moreover, when the chief reliance has been placed upon this remedy, its use has often prevented that resort to astringents and stimulants which would have so greatly increased the chances against the patient.

What then shall be done by the physician?

In the first place the patient should be placed in bed, to procure that equalization of the circulation which is favored by the warmth of the couch, and is most favorable to the action of the secernent glands. As remedial agents, there can be nothing better in the stage of cholerine than the mildest of mercurials, alternated with the sulphuric acid lemonade. But if the disease advance to the stage of prostration which threatens collapse, mercurials, astringents and opiates should be abandoned. We must now favor evacuation, relieve pain, and control the circulation. Give one large dose of castor oil, to remove all offensive matter from the bowels, and if there is great distress and prostration without evacuation, give soda powders, largely diluted, to prevent irritation, which, by the action of their saline ingredients, will serve to provoke the discharge of water from

* This is disputed by some who assert that the more active the vomiting and purging in a case of cholera, the more certainly fatal will be the result. Yes, if the ordinary treatment is conducted with an activity proportioned to the urgency of these symptoms; for, as I have shown, the danger of the patient is proportioned to the degree of success which attends the hindrance of these vicarious processes of elimination, and it is in precisely this class of cases that the largest doses of opium, acetate of lead, etc., followed by the most copious libations of brandy, are usually employed.

the blood, and by their effervescence will tend to allay the irritability of the gastric surfaces. If perspiration is deficient, give warm mint-tea containing a drachm or two of sulphuric ether. To relieve dyspnœa, to allay the pain occasioned by muscular cramp, and to overcome that irritability for which opium is usually administered, give chloroform by inhalation and by the mouth. The effect of chloroform must be very carefully watched, because, if carried too far, the brain will become congested, the blood will become still more deprived of oxygen, and by excessive relaxation of the pulmonary arterioles, combined with the stimulation of the heart, which is one of the primary effects of chloroform, too great a supply of vitiated blood may be discharged upon the tissues, which are already in peril from contact with the poisonous fluid. Chloroform will be most serviceable when collapse comes on early, (as the result in great measure of mechanical impediment to the pulmonary circulation,) before the vitality of the tissues has been so far depressed that they feel but slightly the need of oxygen to sustain the processes of nutrition. In this stage the patient will be relieved by chloroform, very much as a criminal, half-strangled by a hangman's cord, is relieved by the knife which severs the noose around his neck. Chloroform may relieve, but unfortunately, it cannot cure, for the blood of the cholera patient contains a poison which will destroy the life of the tissues, and which can neither be neutralized nor eliminated by agents like chloroform, bleeding, tartar emetic, etc., which simply relax spasm or allay irritation. All these agents have been found useful, but, except so far as they favor evacuation, their good effects are restricted to the single property of controlling spasm and irritability. Their secondary effects are useless if not positively injurious, and their employment should be restricted to those cases where the phenomena which result from impeded circulation, have with them associated a degree of dyspnœa and distress which indicates that the vitality of the tissues is not yet so far overpowered that the want of oxygen is no longer experienced by the system. When vitality has ebbed so low that the cessation of the pulse awakens no responsive respiratory effort, the use of chloroform will be attended with more danger than accrues from bleeding and tartar emetic.

But in this stage, when collapse follows the season of prostration in the ordinary course of the disease, these active agents should seldom be employed. The stage of collapse is a recuperative stage. It is not syncope, but, like the syncope which saves the wounded man by arresting hemorrhage, its design is to save by relieving as far as possible the enfeebled system from contact with the poison which still remains. It is not so much the result of diminished fluidity of the blood, as it is the result of an intolerance of that blood by the system. Hence, our treatment in this stage must tend as little as possible to perturb the operations of nature. Stimulants and astringents are contra-indicated, for the reasons which have been already detailed; opiates, for the same reason, as well as for their tendency to promote cerebral congestion and the retention of excrementitious substances in the blood. Chloroform and tartar emetic are too dangerous. Bleeding may prove beneficial by still farther evacuation of the poisonous agent, and by adding vigor to the heart through depletion of its cavities, but it is liable to the risk of adding syncope to collapse. There remains then little to do but to persist in carrying out the fourth indication for treatment, viz: to supply the materials for the restoration of a healthy circulation. Considering again the nature of the evacuations in cholera, we find that they consist chiefly of the water of the blood. This is discharged from the capillaries, and is recruited from the fluids of the body, occasioning that frightful emaciation so characteristic of the disease, and producing the fearful thirst which consumes the patient. Here is the plainest indication for treatment: Give water freely. The greater portion will be vomited, but some will be retained, and by the process of endosmosis will enter the veins to replace the ejected serum.* During the earlier stages of the process of evacuation, the exosmosis of the serous portion of the blood will be favored by the administration of water, in which is dissolved a quantity of salt somewhat exceed-

* Water should be imbibed slowly, to avoid the tumultuous discharges which may result from suddenly overloading the stomach with large draughts of fluid. Hence the advantage of sucking ice.

ing the proportion of the saline constituents of the blood. During the later stages of the disease, when the blood has been largely drained of its water, and when, consequently, its replenishment is desirable, endosmosis should be favored by the administration of water containing a smaller proportion of saline ingredients than exist in the blood. In this way the renewal of the blood will be promoted in a manner far less dangerous than by the method of injecting saline solutions into the veins. As soon as reaction commences, cups should be applied to assist in relieving the engorgement of the kidneys and the liver, and as soon as urine begins to flow, the elimination of urea from the blood must be encouraged. The hepatic secretion will soon be re-established, and the patient may be cared for in accordance with the general principles which govern the treatment of the various conditions which grow out of the different forms of reaction.

Such, then, is the theory of the pathology and treatment of cholera, derived from a consideration of its symptoms and its morbid appearances. I have said little about the accessories of treatment, such as sinapisms, foot baths, friction, ice, vapor baths, warm baths, etc., because the limits of this paper restrict one to the leading features of the disease and of its treatment, and for the same reason I have not touched upon the pregnant subject of prophylaxis. If we entertain a clear view of the pathology of the disease, we shall at least know how to avoid whatever tends to harm the patient; and while we remain destitute of remedies antidotal to the cause of the disease, the best we can do in our treatment will be, to remove as many as possible of the impediments which hinder the recuperative efforts of nature, while we endeavor to sustain the powers of the sufferer. The mortality of cholera will always be large, and the percentage of deaths will always be greater at the commencement of an epidemic than towards its close, from the fact that those persons whose livers and kidneys have been damaged by previous disease, are, by consequence, pre-eminently susceptible to the invasion of cholera, and are especially liable to fatal complications during its course. If, recognizing these facts, we shall, without wavering or discouragement, continue to apply that treatment which is founded upon the established principles of physiology and pathology, we may confidently expect to see a great reduction in the general average mortality resulting from this terrible pestilence.

Since the completion of the *a priori* argument contained in the preceding pages, my friend, Dr. Marguerat, has placed in my hands the following statistics, from the hospitals of London, Paris and Vienna, which serve to illustrate the practical working of the different theories which have been formed for the guidance of the physician in cholera:

TREATMENT AND MORTALITY OF CHOLERA.

| TREATMENT. | Cases. | Deaths. | Per ct. |
|---|---|---|---|
| Venous injection,. | 90 | 78 | 85.7 |
| STIMULANTS. | | | |
| Brandy, ammonia, turpentine, cajeput oil, etc., | 3055 | 1792 | 58.8 |
| Ditto with ipecac emetics, | 37 | 25 | 67. |
| Ditto with calomel and opium, | 356 | 214 | 60. |
| Ditto with ice, | 58 | 29 | 50. |
| Opium, | 81 | 47 | 58. |
| Calomel and opium, | 196 | 112 | 57.14 |
| Calomel, | 376 | 147 | 36.59 |
| Bleeding, with calomel, opium and antiphlogistics, | 285 | 168 | 59. |
| EMETICS. | | | |
| Ipecac, | 21 | 12 | 57. |
| Ditto with stimulants, | 37 | 25 | 67. |
| Ditto with bleeding and quinine, | 161 | 81 | 50. |
| Ditto with bleeding alone, | 222 | 104 | 47. |
| Ditto with moderate warmth, | 281 | 98 | 34.9 |
| Salt with cold water,* | 607 | 112 | 20. |
| Tartar emetic, | 21 | 4 | 19. |
| Ice with stimulants, | 58 | 29 | 50. |
| Ice alone, | 142 | 43 | 30. |
| SALINES. | | | |
| Greville St. Hospital combination of salts,† | 107 | 15 | 14. |
| Dr. Stevens' combination of salts, | 88 | 67 | 76.3 |
| MISCELLANEOUS, | 17 | 8 | 47.6 |
| Total, | 6296 | 3210 | 51. |

*London Medical Times*, vol. 19, p. 106.

* The salt emetic treatment consisted in the administration of about two tablespoonfuls of common salt in from four to eight ounces of cold water, every quarter of an hour, until vomiting is produced; then cold water in large draughts.

† The saline treatment consisted in the administration, every quarter of an hour, of sodæ carb. scr. ij, sodii chlorid. dr. ij, potass. chlorat. gr. viij, aquæ q. s. pro. haust. The patient was also placed in a hot saline bath, at 120 degrees, in which 14 lbs. of salt were dissolved. Cold water was drank *ad libitum*. See "The Medical Times," vol. 19, p. 89.

In reviewing these figures, it should not be forgotten that the mortality in private practice is much less than it is in public hospitals, to which are carried the worst cases from the dregs of the population. Keeping this fact in mind, one cannot fail to perceive that the tendencies of the disease are to recovery. The most unfavorable circumstances cannot prevent the convalescence of a large proportion of those who are attacked; and when the treatment is conducted in accordance with the settled principles that control the general therapeutics of all other diseases, the mortality is comparatively trifling.

